# Effect of 3′,4′-Dihydroxyflavonol Eye Drops in a Rat Model of Dispase-Induced Proliferative Vitreoretinopathy

**DOI:** 10.3390/antiox14121414

**Published:** 2025-11-27

**Authors:** Elsa C. Chan, Cheng Zeng, Chi D. Luu, Carla J. Abbott, Nicholas T. Chan, Keshava K. Datta, Nicholas Williamson, Penelope J. Allen, Jennifer C. Fan Gaskin

**Affiliations:** 1 Centre for Eye Research Australia, Royal Victorian Eye and Ear Hospital, Melbourne, VIC 3002, Australia; elsa.chan@unimelb.edu.au (E.C.C.); chengzeng0909@gmail.com (C.Z.); cluu@unimelb.edu.au (C.D.L.); c.abbott@unimelb.edu.au (C.J.A.); pjallen@melbourneretina.com.au (P.J.A.); 2 Ophthalmology, Department of Surgery, University of Melbourne, Melbourne, VIC 3002, Australia; 3 Department of Medicine, University of Melbourne, St Vincent’s Hospital, Fitzroy, VIC 3065, Australia; 4 The Bio21 Mass Spectrometry and Proteomics Facility, The University of Melbourne, Parkville, VIC 3010, Australia; 5 Royal Victorian Eye and Ear Hospital, East Melbourne, Melbourne, VIC 3002, Australia

**Keywords:** ARPE-19, 3′,4′-dihydroxyflavonol, proliferative vitreoretinopathy, proteomics, rat model of dispase-induced PVR

## Abstract

(1) Background: Proliferative vitreoretinopathy (PVR) is the most common cause of failure in retinal detachment surgery and often leads to blindness. Oxidative stress is known to contribute to scar formation; therefore, reducing oxidative stress may protect against PVR development. This study investigated the therapeutic effects of the antioxidant 3′,4′-dihydroxyflavonol (DiOHF) in two preclinical models of PVR. (2) Methods: A retinal pigment epithelial cell line (ARPE-19) was used to investigate the anti-fibrotic effects of DiOHF. PVR was induced in one eye of each animal using dispase. Animals then received either vehicle or DiOHF eye drops in both eyes for 28 days. Eyes were harvested for mass spectrometry to perform proteomic analysis or to quantify tissue accumulation of DiOHF. Proteomic analysis was also performed in ARPE to validate these findings. (3) Results: In DiOHF-treated eyes with induced PVR, proteomic profiles showed reduced fibrosis, inflammation, cell migration, and oxidative stress compared with vehicle-treated PVR eyes. The in vitro studies confirmed that DiOHF inhibited wound healing responses, cell contraction, proliferation, and the generation of reactive oxygen species in ARPE-19 cells. Proteomic analysis in ARPE-19 also showed a similar trend. (4) Conclusions: This study provides compelling evidence that DiOHF eye drops offer protective effects against PVR in preclinical models.

## 1. Introduction

Proliferative vitreoretinopathy (PVR) is a serious condition resulting from a complication of retinal detachment or retinal trauma. Fibrous tissues form on the detached retina, preventing the reattachment of the retina, and commonly lead to blindness. Surgical intervention is the only recommended solution; however, repeat surgery is often required and is associated with intrinsic risks and limited success [1]. The development of a non-invasive approach is keenly sought to address the unmet medical need for PVR treatment.

Since PVR occurs due to uncontrolled wound healing responses [2], intervention targeting different stages of wound healing have been investigated as an adjunct treatment following retinal detachment surgery [3], including corticosteroids, anti-VEGF, and anti-proliferative agents such as methotrexate and 5-fluorouracil [3,4]. However, some of these adjunct treatments are toxic antimetabolites or cell-killing compounds (i.e., methotrexate and mitomycin C) and come with significant side effects like corneal ulceration, retinal ischaemia, and scleral perforation [5,6]. Furthermore, the current adjunct therapies require invasive eye injections, and alternatives such as simple eye drops would reduce the risk of treatment burden on patients. As such, an alternative approach such as eye drops would be ideal.

A polyphenolic compound named 3′,4′-dihydroxyflavonol (DiOHF) has been demonstrated by our group to suppress postoperative scarring in an experimental model of glaucoma surgery when given as eye drops [7]. Moreover, DiOHF has been shown to suppress biological processes in human Tenon’s fibroblasts that are known to contribute to postoperative conjunctival fibrosis [8]. Based on these preclinical findings, we hypothesise that DiOHF would confer beneficial effects in PVR. Intraocular diseases have the advantage of being easily accessible through local therapy and could eliminate any undesirable systemic side effects. Medication in an eye drop form is non-invasive, easy to administer, and potentially titratable depending on stage and severity of disease, offering numerous potential advantages over systemic therapy or even intraocular injections. To test this hypothesis, we evaluated the effects of DiOHF eye drops in a rat model of dispase-induced PVR [4] by comparing the proteomic profiles between vehicle control and PVR groups. Using a cell line of retinal pigment epithelial cells ARPE-19, we also verified whether DiOHF affected the biological processes contributing to scarring.

## 2. Materials and Methods

### 2.1. Cell Culture and Treatment with Pharmacological Agents

A cell line of retinal pigment epithelial cells ARPE-19 was purchased from ATCC (Manassas, VA, USA). Cells were cultured in Dulbecco’s Modified Eagle Medium (DMEM)/F12 (Merck, Bayswater, VIC, Australia) supplemented with fetal calf serum (10%; Bovogen, Keilor East, VIC, Australia), penicillin-streptomycin (100 U/mL; 100 μg/mL, Merck) at 37 °C in 5% CO_2_. Cells were seeded in 6-well, 24-well, 48-well, and 96-well plates and were serum-starved prior to stimulation with TGFβ1 (10 ng/mL, Merck) + PDGF-BB (1 ng/mL; Merck). To examine the inhibitory effects of DiOHF (10 μM), cells were pre-treated 1 h prior to TGFβ1 and PDGF-BB treatment for up to 48 h, depending on the endpoint assays as described herein: the determination of total reactive oxygen species (ROS), expression of marker of epithelial–mesenchymal (EMT), wound healing assay, cell proliferation, and collagen gel contraction, as previously described [8]. Cells were also seeded to ~80% confluency in a 60 mm culture dish and assigned to three treatment groups: (1) control; (2) TGFβ1 (10 ng/mL; Merck) + PDGF-BB (1 ng/mL; Merck) + DMSO (0.1%); (3) TGFβ1 (10 ng/mL) + PDGF-BB (1 ng/mL) + DiOHF (10 μM). DMSO was a solvent to solubilise DiOHF. Cells were harvested at 48 h and prepared for mass spectrometry as outlined under “Proteomic Analysis by Mass Spectrometry”. An overview of experiments conducted in the ARPE-19 cell line and in the rat model is presented in Supplementary Figure S1.

### 2.2. Determination of Total ROS and Expression of N-Cadherin in ARPE-19

The level of ROS was determined by a 2′,7′-dichlorofluorescin diacetate (DCFDA) assay (2.5 μM, ab113851; Abcam, Melbourne, VIC, Australia) in ARPE-19 cells according to the manufacturer’s instructions as previously described [8]. The treatment groups are control and TGFβ1 + PDGF + DiOHF, given either 1 h before (DiOHF (1 h)) or at the same time as the addition of TGFβ1 + PDGF. Cells (2.5 × 10^4^ cells per well) were seeded in a black clear-bottom plate for the DCFDA assay. To determine the expression of N-cadherin, ARPE-19 was seeded in a 48-well plate (5 × 10^4^ cells per well). Cells were harvested at 48 h for the evaluation of N-cadherin with ELISA according to the manufacturer’s instructions (ab254512, Abcam).

### 2.3. Wound Healing Assay and Cell Proliferation

ARPE-19 cells were seeded at 10^6^ cells per well. A wound in the shape of a cross was created with a sterile pipette tip. Four non-overlapping images (2048 × 1532 pixels) of the wound were captured from each well under 4× magnification at 0 and 24 h. The same wound area was selected for measurement at 0 and 24 h. The distance between the wound was measured from three different regions of the wound using Fiji [9]. Cell migration at 24 h was expressed as percentage of wound recovered compared with the start of the experiment (t = 0 h). Cells were seeded in a 48-well plate (16,000 cells per well) to assess cell proliferation. At 48 h, DAPI-stained nuclei were determined from three images captured at 10× magnification (Nikon inverted microscope, Rhodes, NSW, Australia). Averages from the three images were determined as a measure of cell proliferation at 48 h after stimulation of TGFβ1 and PDGF-BB.

### 2.4. Collagen Gel Assay

The contractility of ARPE-19 was assessed by a collagen gel assay. A collagen solution was prepared in DMEM/F12 with 2% FBS containing NaOH, 10× phosphate-buffered saline, and rat tail collagen I (1 mg/mL, A1048301; Thermo Fisher Scientific, Scoresby, VIC, Australia) with 500,000 cells/mL. TGFβ1 was added to the collagen solution before seeding in 24-well plates (0.35 mL/well). Collagen was polymerised at 37 °C and then detached from the well edges. Gel was floated in a serum-free medium (0.5 mL) with DiOHF (10 μM) or a vehicle and imaged. The gel area was quantified using Fiji [9]. Values are expressed as the percentage of the area of the control gel.

### 2.5. Rat Model of PVR

All surgical procedures were performed according to the guidelines of St. Vincent’s Hospital Melbourne Animal Ethics Committee (Ethics no. AEC 017/24). A procedure to induce PVR was performed to the right eye of 12 male Sprague Dawley rats (250–350 g, supplied by the University of Melbourne Biomedical Sciences Facility) under general anaesthesia (xylazine, 10 mg/kg, Troy Animal Healthcare, Glendenning, NSW, Australia and ketamine, 75 mg/kg, Ceva Animal Health, Glenorie, NSW, Australia; intraperitoneal). The procedure of dispase-induced PVR was based on established techniques by Uslubas et al. [4]. Intravitreal injections of 0.5 μL of dispase (Merck) was injected with a 30 G needle. The contralateral eye was not treated and was used as the control. After dispase injection, rats were then assigned to one of three treatment groups (N = 4 rats/treatment): vehicle, DiOHF (Merck), and methotrexate. DMSO was used as a solvent for DiOHF. Vehicle (0.1% DMSO in 40% g-cyclodextrin; Chem-supply, Port Adelaide, SA, Australia) and DiOHF (100 μM in 40% g-cyclodextrin) eye drops were administered to the dispase-injected eyes in their respective groups three times each day for 28 days. Methotrexate was administered as a single bolus intravitreal injection (0.5 μL) in the dispase-injected eyes of the methotrexate group after dispase injection. The contralateral control eyes were treated with DiOHF at 10, 30, and 100 μM three times a day for 28 days to quantify the amount of DiOHF in the retina and vitreous humour. Fundus imaging was performed on dispase-injected eyes before and after dispase injection and at the time of tissue harvest on day 28. Optical coherence tomography (OCT) and near-infrared (NIR) imaging was also performed on both non-injected and dispase-injected eyes on day 28. Investigators performing fundus and OCT imaging were masked to the treatment status of the animals. All 12 animals were included from the beginning until the end of the experiments. Exclusion of animals due to illness/injury was not required.

### 2.6. Sample Preparation for Proteomic Analysis by Mass Spectrometry

Animals were euthanised with Lethabarb (Virbac, Milperra, NSW, Australia; 1 mL/kg, intraperitoneal) and eyes were enucleated. Vitreous humours and retinas were dissected from either control or dispase-injected eyes and they were lysed in sodium dodecyl sulphate buffer (5% SDS buffer in 50 mM triethyl ammonium bicarbonate; Merck). The protein concentration was determined with a BCA protein assay as per the manufacturer’s instructions (ThermoFischer Scientific). Samples were then prepared for mass spectrometry based on the S-Trap™ micro spin column digestion protocol specified by the supplier (Protifi, Fairport, NY, USA). In brief, an equal amount of protein (11.5 mg) from all samples was reduced by Tris(2-carboxyethyl) phosphine (5 mM final concentration, Merck) at 55 °C for 15 min. Methyl methanethiosulphonate (20 mM, Merck) was used to alkylate disulphide bonds for 10 min at room temperature. Phosphoric acid (2.5% final concentration, Merck) was then used to denature the protein before loading into S-Trap™ micro spin column for peptide extraction. Trypsin (Trypsin/Lys-C Protease Mix, Thermo Scientific) was added at a ratio of 1:10 (enzyme-to-substrate) and incubated at 37 °C overnight to facilitate digestion. Digested samples were eluted three times in the order of triethyl ammonium bicarbonate (50 mM), formic acid (0.2%), and acetonitrile (50%; Merck). Pooled eluates were dried in a speedvac and resuspended in aqueous buffer (2% acetonitrile, 0.05% trifluoroacetic acid), and LC-MS/MS was carried out. Investigators performing mass spectrometry data acquisition and analysis were masked to the treatment status of the animals.

### 2.7. Mass Spectrometry Data Acquisition

An Orbitrap Eclipse mass spectrometer (ThermoFisher Scientific, San Jose, CA, USA) and Orbitrap Exploris 480 mass spectrometer (ThermoFisher Scientific, San Jose, CA, USA) were utilised to acquire LC-MS/MS data for the ARPE cell line and rat proteome, respectively. Both mass spectrometers were interfaced with Ultimate-3000 UHPLCs (ThermoFisher Scientific, San Jose, CA, USA). LC and MS settings for both sample types were identical: Peptides were first loaded onto an Acclaim™ PepMap™ C18 trap column (ThermoFisher Scientific, Bremen, Germany) at 5 µL/min and washed for 6 min. The trap was then brought in line with the analytical column, which was a 50 cm PepMap™ C18 column (ThermoFisher Scientific, Bremen, Germany). The peptides were resolved at a flow rate of 300 nL/min. The solvent system consisted of solvent A (0.1% formic acid, 5% DMSO, in water) and solvent B (0.1% formic acid, 5% DMSO in acetonitrile). The gradient consisted of a linear increase of solvent B from 3% to 25% over 76 min and a ramp up of 25% to 40% over 4 min and 40% to 80% over a minute. The flow was held at 80% B for 3 min to wash the column. Flow was dropped to 3% solvent B and the column was equilibrated for 5 min. The total runtime was 95 min.

The mass spectrometer was operated on a data-independent mode. The instrument settings were: MS1 resolution—120,000; mass range—350–1400 *m*/*z*; normalised AGC Target—250%; maximum injection time 50 ms; MS2: isolation window—13.7 *m*/*z*, leading to 50 scan events; normalised HCD collision energy—30%; normalised AGC target—2000%; maximum injection time—55 ms.

### 2.8. Mass Spectrometry Data Processing

Mass spectrometry-derived raw files of the ARPE cell line and rat were searched against the Homo sapiens and Rattus norvegicus protein databases, respectively. In both cases, the UniProt canonical protein database (one protein sequence per gene) was utilised and database searches were carried out using Spectronaut (Version 19.4) (Biognosys). The search settings for both datasets were identical—2 missed cleavages allowed, carbamidomethylation of cysteine set as fixed modification and acetylation of the protein N-terminus, and oxidation of methionine set as variable modifications. False discovery rate (FDR) was set to 1% at the peptide spectrum match, peptide, as well as protein groups.

### 2.9. Quantification of DiOHF in Ocular Tissues

After 28 days of DiOHF eye drops in control eyes, specimens of retinas and vitreous and aqueous humours were obtained and prepared for mass spectrometry. Quantification was determined with LC-MS performed by the HMS Trust Analytical Laboratory, Monash Institute of Pharmaceutical Science (Parkville, VIC, Australia).

### 2.10. Data Presentation and Analysis

The mass spectrometry results were analysed with Spectronaut^®^ 19.0 (Biognosys, US) with the treatment groups unmasked. Mass spectrometry-derived raw files were searched against the UniProt Homo sapiens canonical protein database (one protein sequence per gene). The protein profile was visualised using volcano plots generated in Spectronaut Post-Analysis. Protein–Protein interactions were analysed by STRING database version 12.0, and the plots are presented [10]. Data are presented as the mean ± SD and were analysed by a one-way analysis of variance (ANOVA) with post hoc (Sidak’s multiple comparisons tests; Prism 10.0, GraphPad, Boston, MA, USA). A *p*-value of <0.05 is considered statistically significant.

## 3. Results

### 3.1. Effect of DiOHF on Cellular Responses Contributing to PVR Injury

**Figure 1:**
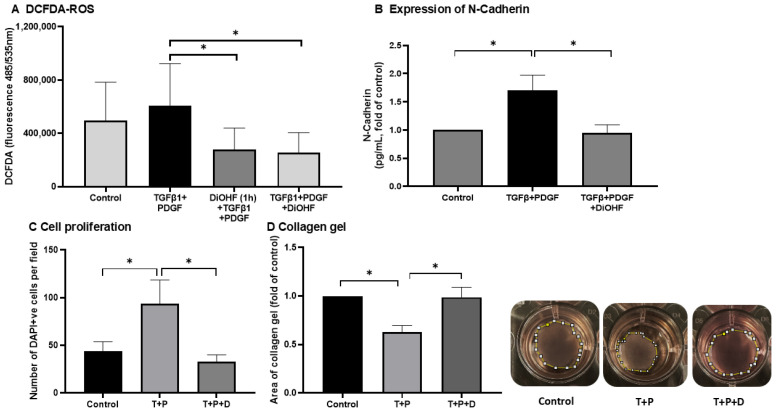
Effect of DiOHF (10 μM) on the stimulatory effects of TGFβ1 (T, 10 ng/mL) and PDGF-BB (P, 1 ng/mL) in ARPE-19. (**A**) DCF fluorescence as a measure of ROS generation. DiOHF, given either 1 h before (DiOHF (1 h) + TGFβ1 + PDGF) or at the same time as TGFβ1 + PDGF (TGFβ1 + PDGF + DiOHF), significantly suppressed ROS generation (N = 7). (**B**) Expression of N-cadherin was determined by ELISA at 48 h after treatment (N = 6). (**C**) Cell proliferation was assessed by counting the number of DAPI +ve positive nuclei 48 h after treatment (N = 5). (**D**) The area of collagen gel embedded with ARPE-19 was measured on day 5 after treatment. The gel is outlined by the dashed line (N = 5). * *p* < 0.05 from a one-way ANOVA with post hoc test.

In this study, we investigated whether DiOHF (10 μM) would affect the stimulatory effect of pro-fibrotic factors TGFβ1 and PDGF-BB in ARPE-19. Both fibrotic factors were used to model PVR scarring in ARPE-19. We evaluated the antioxidant property of DiOHF by assessing the total ROS production with the cell-permeable fluorescent dye DCFDA, which is subsequently oxidised by ROS into DCF. As shown in Figure 1A, the level of DCF fluorescence was reduced by DiOHF when either applied 1 h before or at the same time as TGFβ1 + PDGF-BB. The small induction of TGFβ1 + PDGF-BB on ROS generation suggested that ROS could be relatively high at the basal level (Figure 1A). DiOHF also inhibited a stimulatory effect of TGFβ1 + PDGF-BB on cell proliferation (Figure 1C). Contraction of ARPE-19 was evaluated by suspending the cells in a collagen gel. TGFβ1 + PDGF-BB significantly reduced the area of the collagen gel on day 5 (N = 5; *p* < 0.01; Figure 1D). A reduction in gel area indicated gel contraction, as depicted in Figure 1D. DiOHF (10 μM) significantly prevented the stimulatory effect of TGFβ1 + PDGF-DD on cell contraction (Figure 1D).

### 3.2. Effect of DiOHF on Wound Healing in ARPE-19

**Figure 2:**
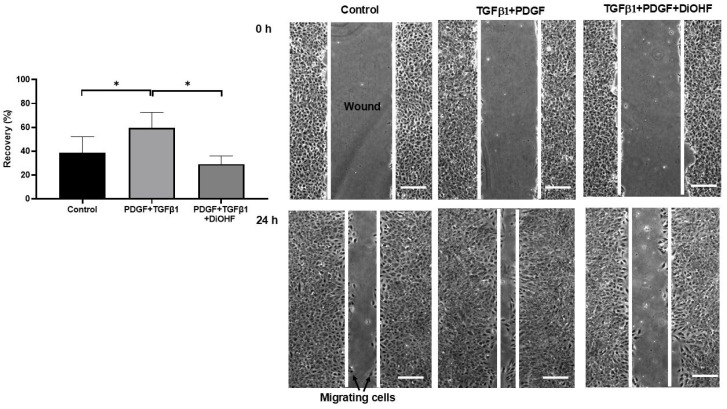
Wound healing responses in ARPE-19. Quantitative measures of recovery following a wound created on a monolayer of cells. Representative images of a wound captured at 0 and 24 h after the addition of TGFβ1 (10 ng/mL) and PDGF (PDGF-BB, 1 ng/mL) with and without pre-treatment of DiOHF (10 μM). * *p* < 0.05 from a one-way ANOVA test with N = 5. Scale bar = 200 μm.

We also evaluated the effect of DiOHF on cell migration in a wound healing assay. DiOHF inhibited the migration of cells to the wound at 24 h following the addition of TGFβ1 and PDGF-BB (Figure 2).

### 3.3. Accumulation of DiOHF

As shown in Table 1, DiOHF was undetectable in either retina/vitreous or aqueous humours from rat eyes treated with three sessions of DiOHF eye drops in 10 μM for 28 days. DiOHF was detected in both 30 and 100 μM groups, and the accumulation appears to be dose-dependent.

### 3.4. In Vivo Observations of Treated Eyes

Immediately after dispase injection into rat eyes, retinal bleeding began, and fibrosis formation followed later as expected. On fundus photography, there were no observed in vivo differences in the amount of retinal bleeding noted between the three treatment groups (vehicle, DiOHF, and methotrexate) at any timepoint (Supplementary Figure S2). There was extensive bleeding immediately after dispase injection, which had resolved by 28 days. OCT images at 28 days post-dispase injection demonstrated a clear difference between dispase-injected and non-dispase-treated eyes, with fibrosis forming from the optic disc and sitting within the vitreous, while the retina showed signs of swelling and disorganisation near the optic disc (Supplementary Figure S3). However, no convincing observable differences were noted on OCT imaging between the treatment groups (Supplementary Figure S3).

### 3.5. The Digestion Efficiency of Proteins Extracted from Retinas and Vitreous Humours

Trypsin digestion efficiency and specificity was determined by comparing our sample peptides with those following a Spectronaut directDIA in silico digestion of rat retinal tissue. All peptides from our sample were categorised as specific (Supplementary Figure S4). This underscores the high efficiency and specificity of trypsin digestion on our samples.

### 3.6. Protein Identification of Peptides Extracted from Rat Retinas and Vitreous Humours

**Figure 3:**
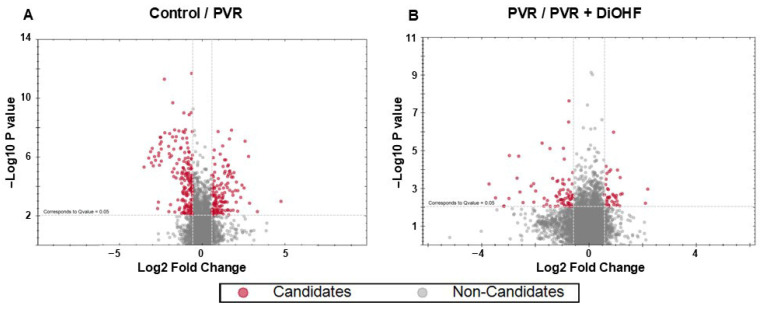
Volcano plots of differentially expressed proteins (DEPs) in the retina. Retinas were exposed to one of 3 treatment conditions: control, PVR, and PVR + DiOHF (N = 4 per condition). Volcano plots of proteins were plotted to compare regulation in (**A**) control/PVR (PVR as compared with the control) and (**B**) PVR/PVR + DiOHF (PVR + DiOHF as compared to PVR). FDR correction was applied with a q-value ≤ 0.05. Upregulated proteins are presented to the right, while downregulated proteins are to the left. Significance in differential regulation was set as log2|fold change| ≥ 0.58, with significantly regulated DEPs being highlighted in red.

Post-analysis of the LC-MS/MS raw data was conducted using Spectronaut directDIA. The post-analysis perspective was used to generate two volcano plots from differentially expressed proteins (DEPs) that were identified in our retina tissue samples (Figure 3). One plot showed DEP regulation from control to PVR (control/PVR), and the second showed DEP regulation from PVR to PVR + DiOHF (PVR/PVR + DiOHF). The volcano plots showed a total of 150 ± 50 DEPs between control and non-control conditions. To highlight significantly regulated DEPs, a false discovery rate (FDR) threshold of q-value ≤ 0.05 was applied. Furthermore, the fold change threshold was set at log2|fold change| ≥ 1.4. Applying these filters revealed 324 significantly regulated DEPs in the control/PVR comparison and 113 in the PVR/PVR + DiOHF comparison.

**Figure 4:**
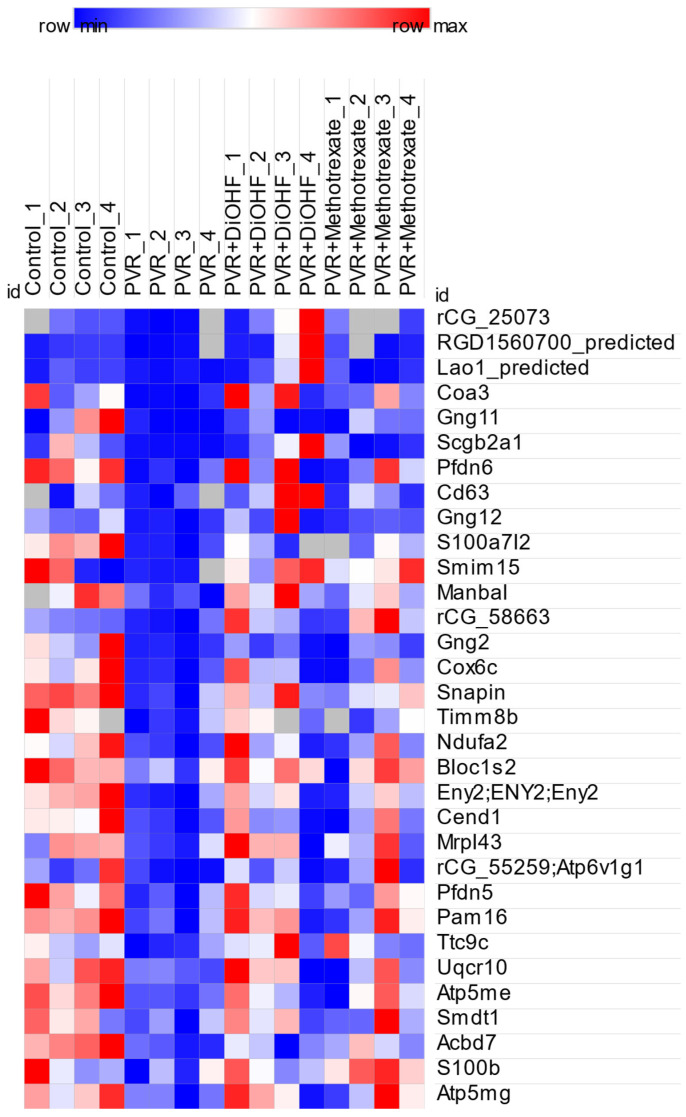
Heat map showing the differential expression of proteins across four experimental conditions in rat retina/vitreous humour: control, proliferative vitreoretinopathy (PVR), PVR + DiOHF, and PVR + methotrexate. Colour intensity reflects relative protein expression levels, with red indicating higher and blue indicating lower expression compared with the mean.

Amongst the significantly regulated DEPs, protein candidates involved in the DiOHF response could be identified. This refers to proteins for which PVR causes differential regulation and DiOHF reverses the regulation. As such, we identified significantly regulated DEPs that were oppositely regulated in the control/PVR comparison and the PVR/PVR + DiOHF comparison, respectively. These included either DEPs that were upregulated in control/PVR, then downregulated in PVR/PVR + DiOHF or downregulated in control/PVR, then upregulated in PVR/PVR + DiOHF. Matching across the groups was based on the DEPs’ gene names, protein descriptions, or UniProt primary accessions. Candidate identification revealed 33 candidate proteins that are shown in Table 2. The differences in regulation across different conditions are shown in a heat map (Figure 4), with all candidates appearing to show less expression in PVR in comparison with PVR and being subsequently upregulated with DiOHF treatment. These 33 proteins were then used for a STRING network analysis, with their respective query items also being shown in Table 2.

The 33 identified candidate proteins had functions related to the three phases of wound healing: inflammation, cell migration and proliferation, and scarring. We also identified proteins with mitochondrial protein complex activity as these may be involved in creating oxidative stress and thus relate to the antioxidant nature of DiOHF. The proteins S100b and Scn5a are related to inflammation [11,12]. Several proteins were involved in regulating cell migration and differentiation. This includes Pfdn6, which is important in microtubule activity, and C18orf32, which was found to activate cell proliferation [13,14]. As for scarring, we identified the candidates Ppt1 and Cend1 that have demonstrated associations with kidney and liver fibrosis scarring, respectively [13,15]. In terms of mitochondrial protein complexes, we found Uqcr10, Coa3, Atp6v1g1, Atp5me, Atp5mg, Timm8b, Pam16, and Smdt1. Furthermore, Lao1 generates H_2_O_2_ and promotes the production of ROS [16].

### 3.7. Protein–Protein Interaction Network Analysis from Candidate Proteins Identified from Retina/Vitreous Humour

**Figure 5:**
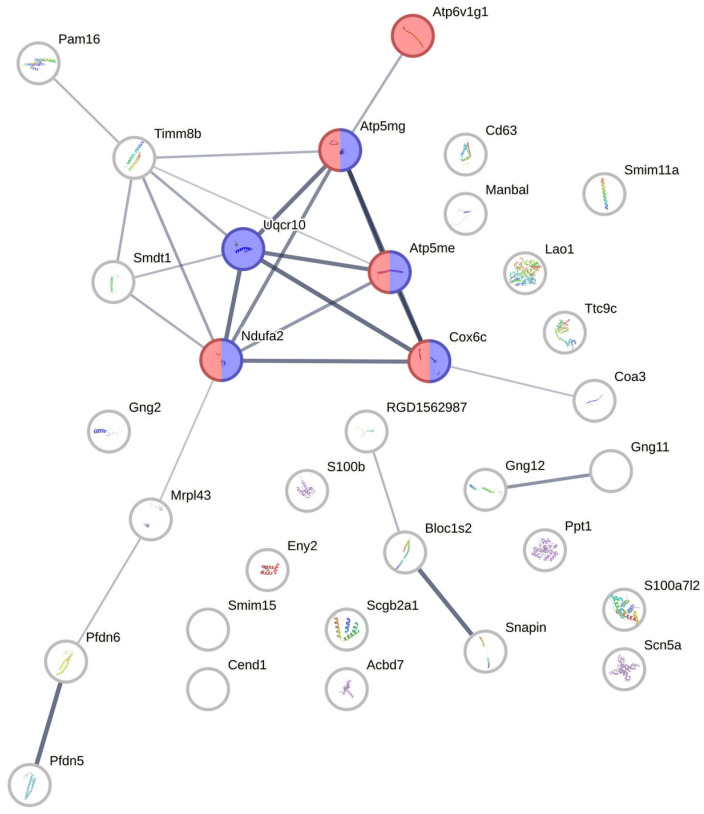
Protein–Protein interaction network containing 33 nodes, constructed from the STRING analysis of the candidate proteins in retina/vitreous humour. Functional enrichment was added. Nodes represent individual proteins. Red nodes (5 out of 33) = “oxidative phosphorylation”. Blue nodes (5 out of 33) = “respiratory chain complex and proton-transporting ATP synthase complex” (electron transport chain). Line thickness indicates interaction confidence.

A protein–protein interaction network between the candidate proteins was constructed using STRING v12.0 (Figure 5). Proteins were queried as shown in Table 2. Functional enrichment analysis was added to the network, with all functions related to the two that were selected to highlight oxidative phosphorylation and the respiratory electron transport chain. The five proteins involved in oxidative phosphorylation were Atp6v1g1, Atp5mg, Atp5me, Cox6c, and Ndufa2. The five proteins involved in the electron transport chain were similar, except that Atp6v1g1 was not highlighted and the additional Uqcr10 was highlighted. Uqcr10 appeared to be a hub gene in the network. The thicker lines between the five nodes also indicate the higher level of confidence of the predicted interactions among them in comparison with other predicted interactions.

### 3.8. Proteomic Analysis on Protein Extracted from ARPE-19

We also conducted proteomic analysis on the ARPE-19 cell line. Injury responses were induced in the cells by the addition of TGFβ1 and PDGF-BB. The procedures for LC-MS/MS and subsequent data analysis were conducted as they were for the retinas. From this analysis, we identified a panel of candidate proteins (Supplementary Figure S5 and Supplementary Table S1) that are identified to be differentially regulated between control-vs-TGFβ1 and TGFβ1-vs- TGFβ1 + DiOHF in ARPE-19 cells. In addition, candidate proteins related to the processes of scarring, inflammation, cell migration and differentiation, and mitochondrial protein complex activity were also identified by searching for related keywords in “GO Molecular Function” and “GO Biological Process” in a Spectronaut GO enrichment analysis. In relation to fibrosis, we identified TGFβ, latent TGFβ binding protein 3 (LTBP3), collagen alpha-2(I) chain (COL1A2), fibrillin-1 (FBN1), integrin-β 5 (ITGB5), procollagen-lysine, 2-oxoglutarate 5-dioxygenase 2 (PLOD2), protein-lysine 6-oxidase (LOX), coiled-coil domain-containing protein 80 (CCDC80), and coagulation factor XIII A chain (F13A1). For inflammation, we identified adenosine deaminase (ADA), extracellular matrix protein 1 (ECM1), lysozyme C (LYZ), keratin type II cytoskeletal 1 (KRT1), apolipoprotein D (APOD), and three S100 proteins (S100A7, S100A8, and S100A9). Related to cell migration and proliferation were fibroblast growth factor 1 (FGF1), ferritin heavy chain (FTH1), and protein S100-A6 (S100A6). As for mitochondrial activity, we identified ATP synthase-coupling factor 6 (ATP5PF), cytochrome c oxidase subunit 7A-related protein (COX7A2L), malate dehydrogenase (MDH2), ferredoxin-2 (FDX2), and two mitochondrial import inner membrane translocases (TIMM10 and TIMM13).

**Figure 6:**
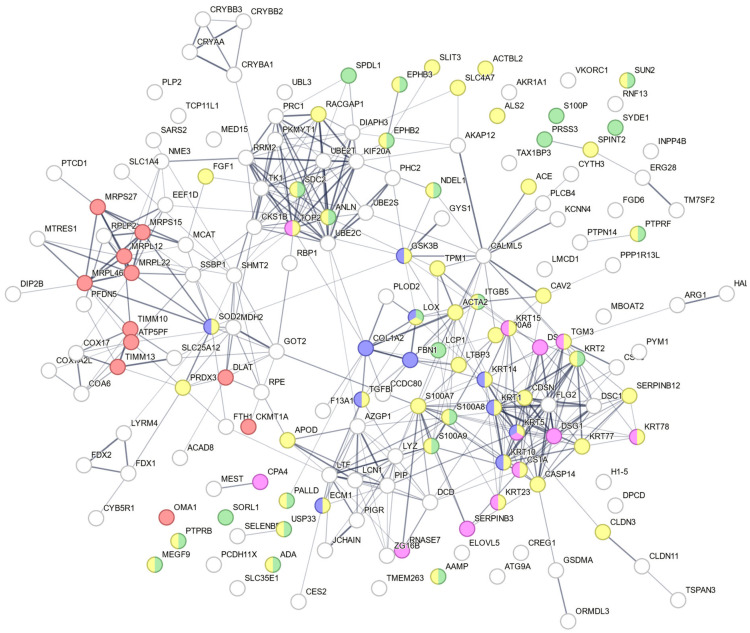
Protein–Protein interaction network containing 173 nodes, constructed from a STRING analysis of the candidate proteins in ARPE-19 cells. Functional enrichment was added. (A) Entire STRING plot: blue nodes = “(2021) the serine proteases dipeptidyl-peptidase 4 and urokinase are key molecules in human and mouse scar formation” (scarring). Pink nodes = “(2022) single-cell RNA-Seq of human oesophageal epithelium in homeostasis and allergic inflammation” (inflammation). Green nodes = “cell migration”. Yellow nodes = “cell differentiation”. Red nodes = “mitochondrial protein complex”. Line thickness indicates interaction confidence.

A protein–protein interaction network generated by the STRING analysis highlighted the interaction of these proteins (Figure 6). They are grouped into five different colours based on their involvement in biological processes: serine proteases dipeptidyl-peptidase 4 and urokinase are key molecules in scar formation (blue nodes), inflammation (pink nodes) cell migration (green nodes), cell differentiation (yellow nodes), and the mitochondrial complex (red nodes).

By scanning for proteins that were differentially regulated in both retinal tissue and ARPE cells (specifically, downregulated during injury then upregulated with DiOHF), we have identified six proteins or protein families that had descriptions with matching retina tissue candidates: cytochrome c oxidase, tetraspanin, S100 proteins, mitochondrial import inner membrane translocases (TIMs), large ribosomal subunit, and prefoldin 5. Cytochrome c oxidase is part of the electron transport chain, with studies showing that increased activity increases wound healing activity [17]. Tetraspanin is abundantly expressed in PVR with roles in wound healing, fibroblast migration, and angiogenesis [18]. Certain TIMs are linked to fibrotic diseases like Timm13 in liver fibrosis [19]. Meanwhile, upregulation of the large ribosomal subunit may increase cell proliferation and differentiation [20]. Lastly, low expression of prefoldin 5 may lead to increased collagen formation [21].

## 4. Discussion

Despite the immense advancement in vitreoretinal surgery in the past few decades (i.e., reduction in gauge and hence sutureless surgery, better viewing systems, better oils, and heavy liquids to aid surgery), PVR remains the biggest challenge to the success of retinal detachment surgery, with a static incidence of 10% of retinal detachments for the past 25 years [22]. Currently, surgery is required to remove the scar tissue that forms in PVR, and more PVR surgery is performed now due to improved surgical techniques. However, the postoperative anatomic success rate after surgery is still at 45–80%, and rates are lower than 60% for severe PVR stages [23]. Alternative pharmacotherapy has been proposed, which involves antimetabolites or cytotoxic compounds like methotrexate and mitomycin C to prevent scar tissue formation. However, the non-specific activity of these compounds can lead to further complications. Thus, safer approaches to PVR treatment are required. Given the promising antiscarring properties of DiOHF in other eye conditions demonstrated by our group previously [7,8], we investigated DiOHF as a potential candidate to treat PVR. The focus of the analysis was a proteomic approach to identify the protein changes in response to PVR following treatment with DiOHF compared with treatment with methotrexate or a vehicle control. DiOHF is a synthetic flavonol that has primarily been investigated for its therapeutic effects in preclinical animal models of cardiovascular diseases [24,25,26]. While the antioxidant property is a main attribute to the protective effect, little is known about the underlying mechanism. Using proteomic analysis, we have identified that DiOHF interferes with the mitochondria oxidative processes to reduce the extent of injury associated with dispase-induced PVR.

Given that the penetration of the ophthalmic formulations administered as eye drops is typically less than 10% [27], we treated the eyes with 10 times higher than the effective anti-fibrotic dose demonstrated in in vitro assays (10 μM). Additionally, we formulated DiOHF with an enhancer known to improve the bioavailability of drugs given as eye drops [28]. DiOHF was undetectable in retinas and vitreous humours from eyes treated with 10 μM but was present in tissues exposed to two higher concentrations (i.e., 30 and 100 μM), with a dose-dependent accumulation. It would not be possible to delineate the exact therapeutic dose of DiOHF based solely on the quantification data as an ocular pharmacokinetic study would be required. However, we have confirmed that DiOHF reached the inner eye compartment to exert an effect on the responses to PVR injury.

We studied changes in the proteome of rat retina tissues and ARPE-19 cells. Our mass spectrometry analysis identified candidate proteins potentially involved in the DiOHF response, specifically those that are differentially regulated from the normal control to PVR and then show opposite regulation following DiOHF treatment. Between the two types of samples we analysed, the STRING analysis revealed that mitochondrial protein complexes were candidates in both samples. In retina tissues, these proteins included ATP synthase (Atp6v1g1, Atp5e, Atp5mg), cytochrome c oxidase (Cox6c), and ubiquinone (Ndufa2). The STRING functional enrichment also determined that these proteins are involved in oxidative phosphorylation, along with complex III (Uqcr10). All these candidate proteins were downregulated during PVR but upregulated with DiOHF treatment. Huang et al. [29] reported similar findings in a transcriptomic study, with oxidative phosphorylation being downregulated in their rat model of PVR. This occurred alongside an increase in oxidative stress. A potential explanation linking impaired oxidative phosphorylation and increased oxidative stress is that in PVR, oxidative stress due to reactive oxygen species (ROS) damages mitochondrial proteins; hence the LC-MS/MS analysis would suggest that they are downregulated. This would also align with the understanding that DiOHF is an oxidative stress-reducing compound that works by scavenging ROS, as shown by the reduction in total ROS in ARPE-19. Other mechanistic studies have also found that the cardioprotective effects of DiOHF were attributed to inhibition of endoplasmic reticulum stress and ROS accumulation [24,25,26]. As such, our proteomic results may provide further evidence that DiOHF reduced PVR by reducing ROS as demonstrated by it increasing mitochondrial protein activity for normal oxidative phosphorylation levels. It is less likely that downregulation of mitochondrial protein complexes caused increased ROS, since inhibiting relevant candidate proteins like complex III is shown to decrease ROS instead [30].

These findings were also observed with mitochondrial proteins of ARPE-19 cells, whereby ATP synthase and CYB5R1 were also downregulated in PVR and upregulated with DiOHF treatment. The evidence regarding the CYB5R family and DiOHF treatment seemingly contradicts the literature, as on the one hand, its activity is associated with the reduction of oxidative stress; we also found that DiOHF prevented the generation of ROS and scavenged ROS under the stimulation of growth factors TGFβ1 and PDGF-BB in ARPE-19. On the other hand, Çelik and Koşar [31] found that other flavonoids inhibited CYB5R proteins, which would contradict both our finding that CYB5R1 was upregulated and that DiOHF should upregulate CYB5R proteins to reduce ROS. It could be that because Çelik and Koşar observed the CYB5 family overall and not CYB5R1 specifically, they did not capture the nuanced effects of flavonoids on CYB5R proteins. Perhaps only some CYB5R family members were inhibited, while those unaffected had strong antioxidant activities. Alternatively, DiOHF may well inhibit CYB5 proteins, resulting in compensatory upregulation in the cells that paradoxically results in reduced oxidative stress. Specific functional studies on this pathway may elucidate the interaction.

TIM proteins were also involved in the DiOHF response, with the treatment upregulating expression. This has been demonstrated to reduce the accumulation of ROS [32]; thus, DiOHF may potentially regulate several mitochondrial proteins to elicit its therapeutic effect.

Beyond mitochondrial proteins, there were several candidate proteins identified in both rat retinas and ARPE-19 cells. We identified S100 proteins as common candidate proteins. The proteomic data revealed S100 proteins to be upregulated with DiOHF treatment; this is possibly due to the inflammatory roles of extracellular S100 proteins [33]. As such, treatment reduced secretion and increased intracellular concentrations of S100 proteins, which was measured as upregulation during the proteomic analysis. The next notable change was the downregulation of the large ribosomal subunit in PVR treatment; this supports the findings of Zhang et al. [34], who demonstrated damage to ribosomes following TGFβ signalling in pulmonary fibrosis. Key components of the large ribosomal subunit, such as L14e, are known to play an important role for protein stability during translation [35]. Conversely, we found that DiOHF subsequently upregulated the large ribosomal subunit, which may enable the production of more stable mitochondrial protein complexes such that they produce fewer ROS, as discussed earlier. The interactions between DiOHF and ribosomes have not been previously studied, but DiOHF treatment has been shown to relieve endoplasmic reticulum stress to preserve endothelial function in diabetic arteries [26]. Both endoplasmic reticulum and ribosomes have been shown to play essential roles in the process of protein secretion in liver tissues [36], hence suggesting that DiOHF may relieve endoplasmic reticulum in a way that would have a positive impact on ribosomes and the associated proteins. The fourth overlapping candidate protein was prefoldin 5, which has been shown to decrease collagen type 1 production in liver fibrosis [21]. Our data found PFDN5 to be downregulated in PVR and upregulated with DiOHF treatment, suggesting that DiOHF treatment reduced collagen accumulation in the scar tissue, thereby reducing fibrosis. As such, this may be another mechanism by which DiOHF treats PVR. However, we found COL1A2 to be upregulated in ARPE-19 cells. This may be an issue related to the cell model. For example, collagen may be required for cell adherence and survival [37], whereas such adherence would not be seen in retinal tissue. Alternatively, the direct exposure of the cells to high concentrations of TGFβ in our PVR model resulted in excessive collagen synthesis [38], whereas in real PVR, many other factors aside from TGFβ are implicated.

Some candidate proteins were neither highlighted on STRING functional enrichment nor overlapping between rat retinas and ARPE-19 cells, yet they are suggested to be important in PVR and DiOHF treatment by other studies. One significant example was AKR1A1 in ARPE-19 cells. A related protein, AKR1B1, has been highlighted as a potential drug target following a proteomic analysis of patient vitreous samples, while genetic association studies find the AKR1B1 gene to be the most prominent risk gene in diabetic retinopathy [39,40]. Mechanistically, overexpression or overactivity of the aldo-keto reductases may increase glucose-to-sorbitol metabolism, which potentially induces oxidative stress [40]. Our analysis revealed that AKR1A1 was upregulated in PVR and downregulated by DiOHF treatment, and while it belongs to the same family of aldo-keto reductases as AKR1B1, we are uncertain if its role in PVR is mechanistically identical. O’Connor et al. [41] explain that both aldo-keto reductases may play roles in glucose metabolism given their expression in kidneys for osmoregulation. Therefore, we might reasonably infer that AKR1A1 plays a similarly important role as AKR1B1 in PVR and its treatment. Other proteins highlighted in other literature include those related to cell adhesion. These are upregulated in PVR as they are important in forming the ECM in a fibrotic scar [42]. We identified tetraspanin in retina tissues, which plays a role in inflammatory cell adhesion [43], though as discussed earlier, it was surprising that tetraspanin was actually upregulated during treatment. In ARPE-19 cells, we identified an integrin (ITGB5) that followed the expected profile of upregulation in PVR followed by downregulation with DiOHF. Furthermore, we found that DiOHF treatment for PVR affected the regulation of serpin proteins (SERPINB3, SERPINB12) and actin (ACTA2) [44]. Overall, the protein responses to PVR and DiOHF treatment in our study corroborate the published literature and may be involved in the drug’s mechanism of action. Further studies exploring the specific effects of DiOHF on these proteins may elucidate their involvement in the DiOHF response.

The animal model of dispase-induced PVR was first established by Frenzel et al. in 1998 [45]. Since then, dispase injection has been one of the most widely used animal models for PVR-related research [4,29]. Dispase is known to induce tissue breakdown, leading to the release of cells and growth factors that initiate PVR development. As we observed using in vivo OCT imaging, dispase causes haemorrhage and fibrosis of a clinically significant scale that resembles several clinical features of PVR [45]. Since growth factors such as TGF and PDGF have been implicated in the progression of PVR [46], we stimulated ARPE-19 with these factors to confirm that DiOHF also inhibited the biological processes contributing to PVR development.

It should be noted that our findings also support the validity of rat retinas as a model for testing DiOHF treatment in PVR. We observed similar regulation profiles in six key proteins involved in PVR and the DiOHF response, indicating that the response in rats is likely similar to that in humans. There were also no contradicting regulation profiles among related proteins of the two models. Our study therefore supports the view that the rat model remains useful to assess PVR and drug responses given the similarities in their proteomic responses. The model’s validity is also supported by similarities in the proteomes of rat and human retinas in glaucoma, suggesting that in ocular pathologies, rat models are useful and valid [47]. Despite the overlaps, there were still differences in the candidate proteins determined for each model. The differences may well be explained by the difference in species. However, the differences are also highly likely explained by the difference between using tissue and cell samples. As the cell samples only revealed the proteomes of RPE cells, only significantly regulated proteins in that cell type were identified. Conversely, when considering the whole retinal tissue, there are more cell types and thus a potentially broader pool of proteins from which Spectronaut directDIA determined as significantly regulated. Given the existence of overlapping proteins and supporting literature, paired with the likelihood that sample type differences played a role in the proteomic differences, we are confident in the rat model of PVR. As such, preclinical testing of DiOHF on rat PVR models remains useful and can help predict the efficacy and safety of the drug before moving into human trials.

There were limitations to our study. Since mass spectrometry tends to detect high-abundance proteins, we may have missed identifying proteins that have very subtle changes. Since we are unable to assess the structure of the retinas retrospectively by histology, changes in the retina morphology may have been overlooked and warrant future histological investigation. We also cannot precisely determine the role of DiOHF on our candidate proteins since proteomic studies do not necessarily prove causal relationships. Instead, our results highlight key proteins that seem to be affected during PVR and subsequent DiOHF treatment, suggesting that further mechanistic studies are required to investigate DiOHF. Furthermore, the in vivo data (fundus photography and OCT) were unable to detect observational differences between treatment groups at 28 days, so the clinical significance is not yet clear.

## 5. Conclusions

A proteomic analysis of DiOHF responses in rat retina and ARPE-19 cell models of PVR revealed key proteins related to fibrotic processes. Importantly, we identified that mitochondrial proteins were upregulated with treatment, aligning with the understanding that DiOHF is an antioxidant that reduces oxidative stress. Furthermore, DiOHF reduced proteins related to fibrosis in general, inflammation, and cell migration and differentiation. Similarities across rat retinas and ARPE-19 cells also indicate that these are good models of PVR. As such, our findings establish a better understanding of DiOHF treatment in valid models. Future studies should investigate the specific role that the candidate proteins play in PVR and DiOHF treatment. Although treatment targeting the retina can be delivered via intravitreal or subretinal administration, ocular injections are inherently associated with risks such as intraocular infection, haemorrhage, and blindness. Therefore, non-invasive approaches such as eye drops have long been pursued for the treatment of retinal diseases. However, bioavailability remains a major challenge due to the need for drugs to penetrate multiple ocular barriers, including the cornea and the anterior and posterior chambers. In light of our findings, eye drops formulated with an enhancer may be a viable drug delivery approach to mitigate the risks associated with treatments for retinal disorders, such as scarring, that would otherwise require multiple intraocular injections.

## Figures and Tables

**Table 1:** Quantification of DiOHF in retina/vitreous and aqueous humours.

Samples	N	Aqueous Humour (ng/g)	Retina/Vitreous (ng/g)
DiOHF (10 μM)	2	Not detected *	Not detected *
DiOHF (30 μM)	3	0.27 ± 0.47	1.19 ± 0.40
DiOHF (100 μM)	3	1.00 ± 0.88	4.58 ± 2.10

* LC-MS has a lower limit of quantification of 0.16 ng/mL.

**Table 2:** Candidate proteins that are significantly differentiated by PVR and affected by DiOHF treatment.

Gene Name	Protein Description	UniProt Primary Accession
Uqcr10	Complex III subunit 9	A0A9K3Y7E2
Coa3	Cytochrome c oxidase assembly factor 3	A6HJA1
Cox6c	Cytochrome c oxidase subunit 6C	A6HR20
Eny2	Transcription and mRNA export factor ENY2	A6HRB0;A6HRB2;A6HRB3
Smdt1	Essential MCU regulator	A6HT67
Ttc9c	Tetratricopeptide repeat domain 9C	A6HZU8
Scgb2a1	Secretoglobin	A6HZZ8
Scn5a	Sodium channel protein	A6I3X4;A6I3X5
Smim15	Small integral membrane protein 15	A6I5J6
Ppt1 *	Palmitoyl-protein thioesterase	A6IS07
Ndufa2	NADH dehydrogenase [ubiquinone] 1 alpha subcomplex subunit 2	A6J329
Timm8b	Mitochondrial import inner membrane translocase TIM8B	A6J4E1
Snapin	Snapin	A6J6M5
Atp6v1g1	V-type proton ATPase subunit G	A6J7Y6;B2GUV5
Bloc1s2	Biogenesis of lysosome-related organelles complex 1 subunit 2	A6JHF0;A6JHF1
Mrpl43	Large ribosomal subunit protein mL43	A6JHH2
S100b	Protein S100	A6JKD9
Smim11	Small integral membrane protein 11	A6JLJ2
Acbd7	Acyl-CoA-binding protein	A6JM23
Gng11	Guanine nucleotide-binding protein subunit γ	A6K2B4
Pam16	Mitochondrial import inner membrane translocase TIM16	A6K4S2
Gng12	Guanine nucleotide-binding protein subunit γ	A6KF41
Gng2	Guanine nucleotide-binding protein subunit γ	A6KKL4
S100a7l2	S100 calcium-binding protein	A6KMS2
Cd63	Tetraspanin	A6KSK3;A6KSK4
C18orf32 **	UPF0729 protein C18orf32 homologue	B1WC88
Pfdn5	Prefoldin 5	B5DFN4
Cend1	C38 protein	B7X6I3
Pfdn6	Prefoldin-β	F7EQJ2
Manbal	Mannosidase, beta A, lysosomal-like	F7ETW1
Lao1	Amine oxidase	F7FCK8
Atp5mg	ATP synthase subunit G	Q6PDU7

UniProt database. The STRING query item was selected after entering the proteins into a STRING search and determining which search query item matched the respective protein. * Identified from a UniProt search of RGD1560700_predicted. ** C18orf32 is the human gene name of the protein. The rat gene name is unavailable as the protein is a homologue of the human “UPF0729 protein C18orf32”.

## Data Availability

The mass spectrometry proteomics data have been deposited to the ProteomeXchange Consortium via the PRIDE [48] partner repository with the dataset identifier PXD068151. The raw in vitro data will be made available by the authors on request.

## Supplementary Materials

The following supporting information can be downloaded at: https://www.mdpi.com/article/10.3390/antiox14121414/s1, Figure S1: An overview of experiments conducted in ARPE-19 and in rats with dispase-induced proliferative vitreoretinopathy injury; Figure S2: Colour fundus images of rat eyes; Figure S3: Optical coherence tomography images of the retina centred on the optic disc at day 28 following induction of PVR. Figure S4: Digestion efficiency distribution following trypsin digestion of the retina tissue samples; Figure S5: Volcano plot of differentially expressed proteins in ARPE-19 cell samples; Table S1: Candidate proteins that are significantly differentiated by TGFβ1 (10 ng/mL) + PDGF-BB (1 ng/mL) and affected by DiOHF treatment in ARPE-19.

## Abbreviations

The following abbreviations are used in this manuscript:

PVR	Proliferative vitreoretinopathy
DiOHF	3′,4′-dihydroxyflavonol
DMEM	Dulbecco’s Modified Eagle Medium
ROS	Reactive oxygen species
EMT	Epithelial–Mesenchymal transition
DCFDA	2′,7′-dichlorofluorescin diacetate
DEP	Differentially expressed proteins
OCT	Optical coherence tomography
LTBP3	Latent TGFβ binding protein 3
COL1A2	Collagen alpha-2(I) chain
FBN1	Fibrillin-1
ITGB5	Integrin-β 5
PLOD2	Procollagen-lysine, 2-oxoglutarate 5-dioxygenase 2
LOX	Protein-lysine 6-oxidase
CCDC80	Coiled-coil domain-containing protein 80
F13A1	Coagulation factor XIII A chain
ADA	Adenosine deaminase
ECM1	Extracellular matrix protein 1
LYZ	Lysozyme C
KRT1	Keratin type II cytoskeletal 1
FGF1	Fibroblast growth factor 1
FTH1	Ferritin heavy chain
ATP5PF	ATP synthase-coupling factor 6
COX7A2L	Cytochrome c oxidase subunit 7A-related protein
MDH2	Malate dehydrogenase
FDX2	Ferredoxin-2
TIMM	Inner membrane translocase
