# Supplementary material for: Effect of 3′,4′-Dihydroxyflavonol Eye Drops in a Rat Model of Dispase-Induced Proliferative Vitreoretinopathy
Source: Antioxidants (Basel). 2025 Nov 27;14(12):1414. doi: 10.3390/antiox14121414 (PMC12729685; doi:10.3390/antiox14121414)
Supplement: Supplementary file 1 [file antioxidants-14-01414-s001.zip › antioxidants-3913871-supplementary.pdf]

Supplementary Figures:

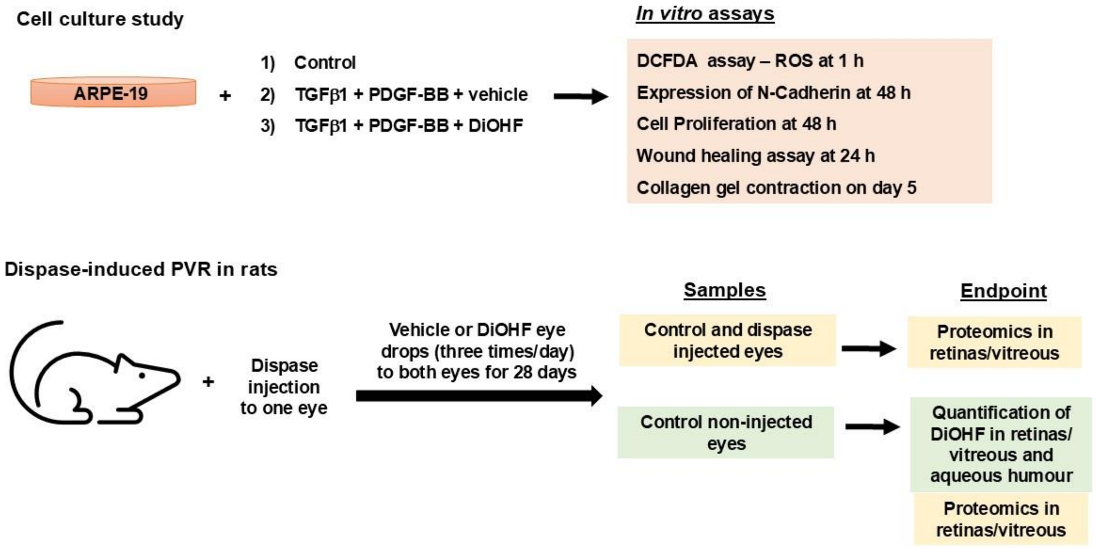

Supplementary Figure S1. An overview of experiments conducted in ARPE-19 and in rats with dispase-induced proliferative vitreoretinopathy injury.

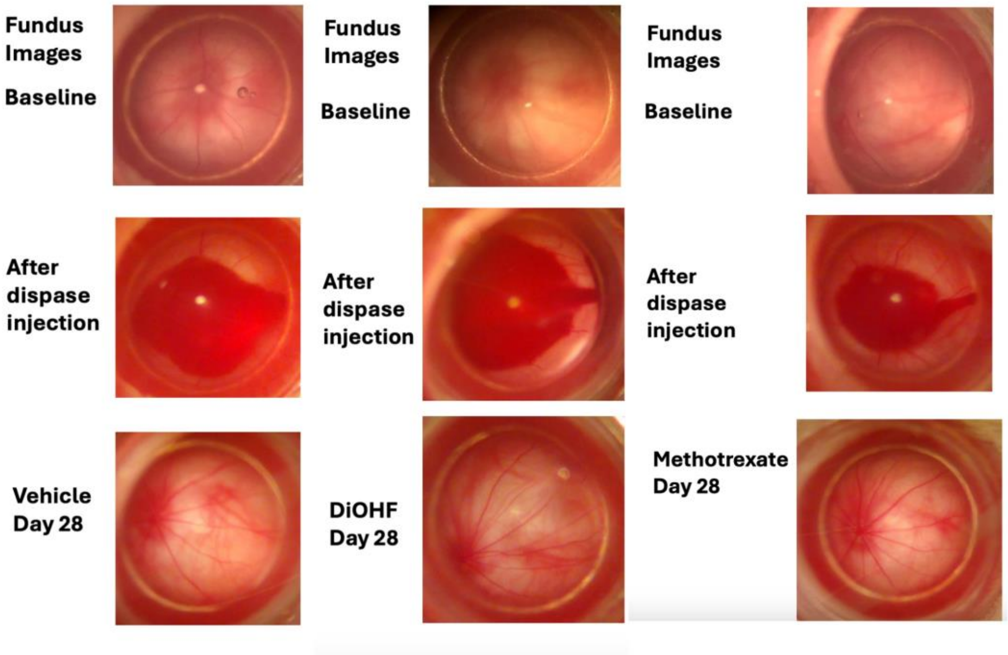

Supplementary Figure S2: Representative colour fundus images of rat eyes at baseline (top row), following induction of PVR by dispase injection (middle row), and at day-28 following treatment with daily vehicle eye drops, daily DiOHF eye drops, and a single injection of methotrexate (bottom row). Retinal and vitreous bleed began immediately after injection and had resolved by day 28 in all groups.

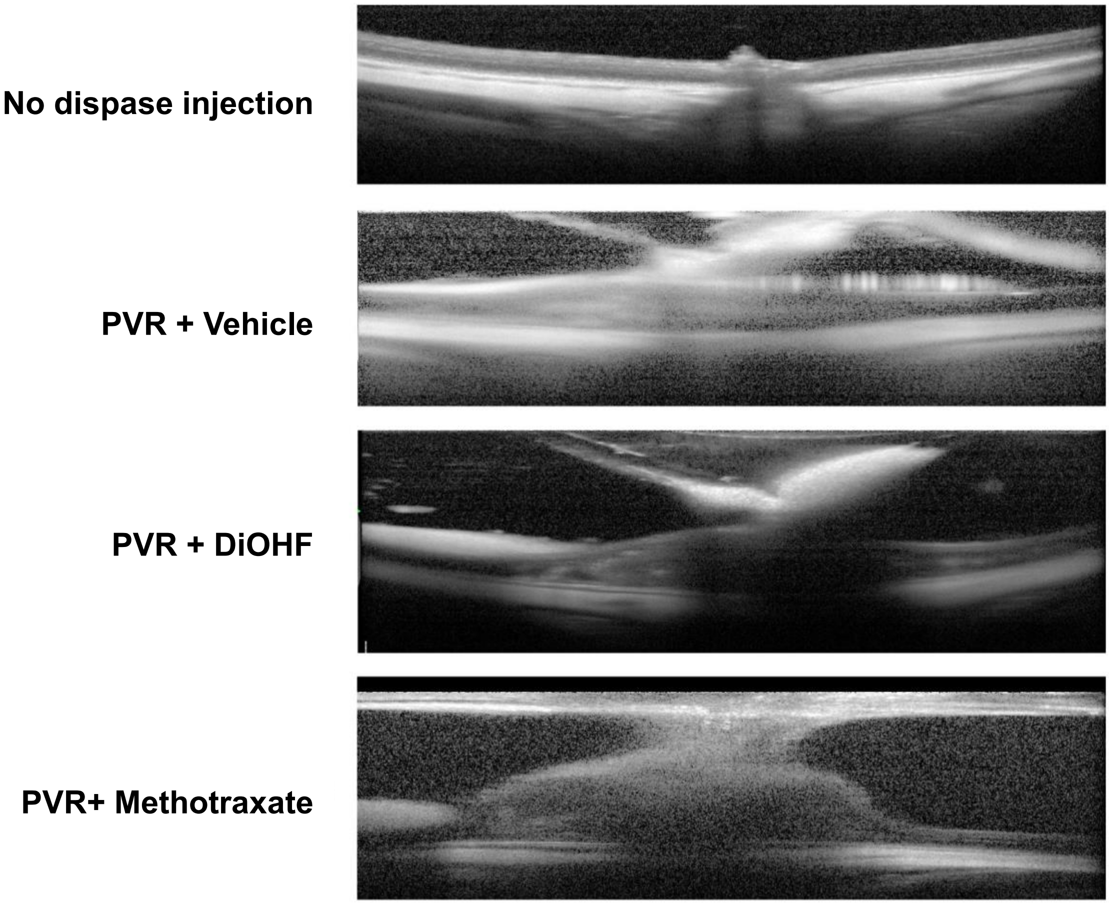

Supplementary Figure S3: Representative optical coherence tomography images of the retina centred on the optic disc at day-28 following induction of PVR in the three groups (vehicle, DiOHF and Methotrexate) compared to control eye (without dispase injection). In all eyes with induced PVR, there is fibrosis present stemming from the optic disc, and sitting within the vitreous. There is also retinal swelling and disorganisation evident near the optic disc in the eyes with induced PVR.

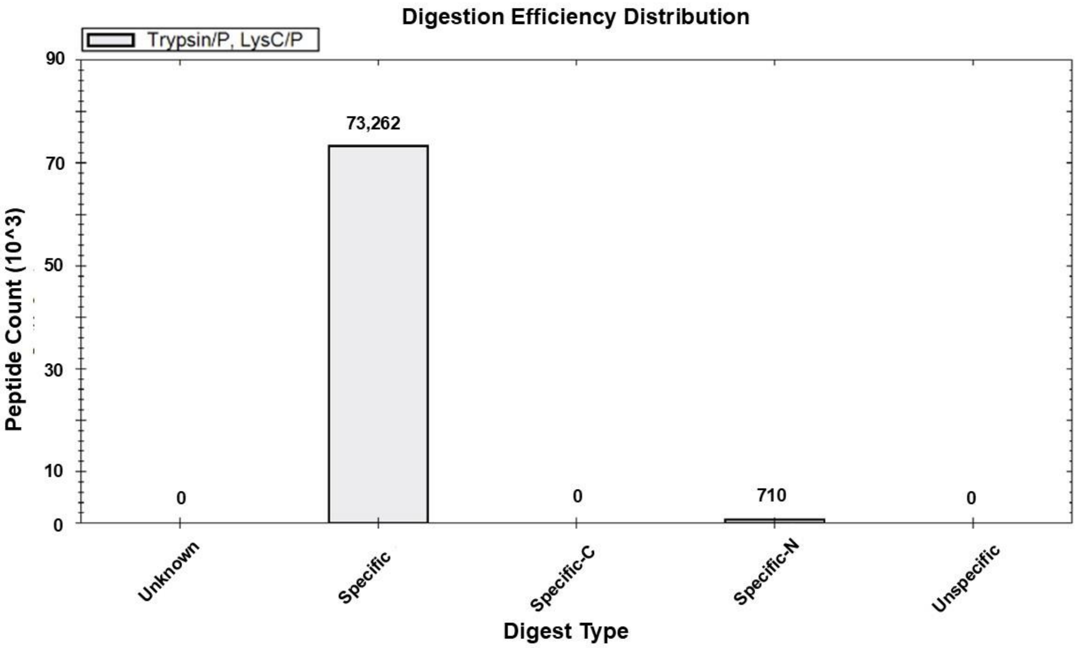

Supplementary Figure S4. Digestion efficiency distribution following trypsin digestion of the retina tissue samples. Following protein extraction and trypsin digestion of rat retina tissue samples, the efficiency and specificity of digestion were determined by comparing sample peptides with *in silico* digestion peptides. Spectronaut directDIA was used to generate *in silico* peptides using the digestion rules "Trypsin/P" and "LysC/P". Upon comparison, sample peptides were categorized into 1 of 5 categories: "Unknown", "Specific", "Specific-C", "Specific-N", and "Unspecific". All 73,792 sample peptides were in either the "Specific" or "Specific-N" categories.

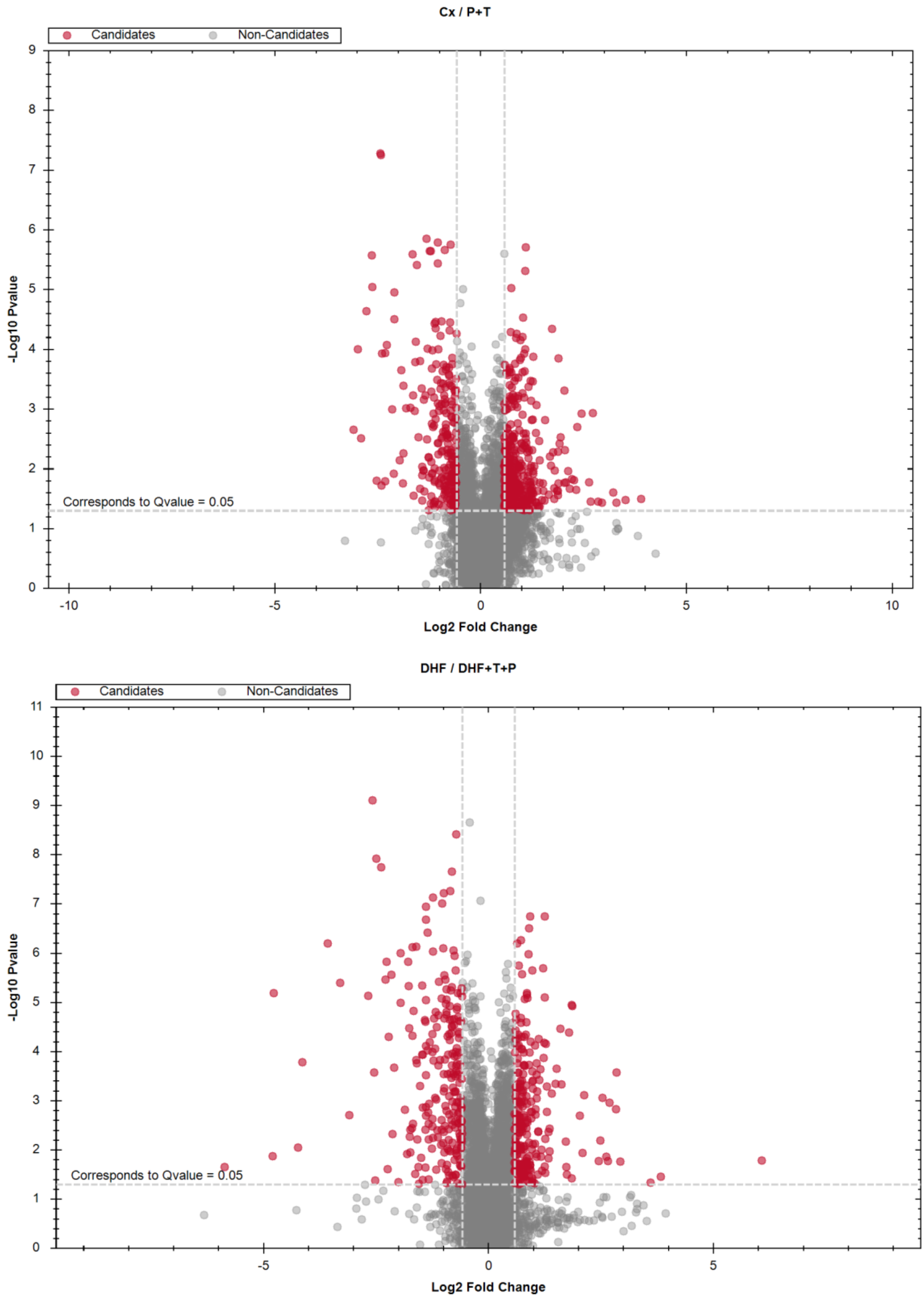

Supplementary Figure S5. Volcano plot of differentially expressed proteins in ARPE-19 cell samples. ARPE-19 cells were exposed to 1 of 3 treatment conditions: control, T+P, and T+P+DIOHF (n=3 per condition). Volcano plots of proteins were plotted to compare regulation in **A)** control / T+P (T+P as compared to control), and **B)** T+P / T+P+DIOHF (T+P+DIOHF as compared to T+P). FDR correction was applied with a q-value ≤ 0.05. Upregulated proteins are to the right, while downregulated proteins are to the left. Significance in differential regulation was set as $\log_2|\text{fold change}| \geq 0.58$, with significantly regulated DEP highlighted in red.

Supplementary Table S1 Candidate proteins that are significantly differentiated by TGFb1 (10 ng/mL+ PDGF-BB (1 ng/mL) and affected by DiOHF treatment in ARPE-19.

Gene name	Protein description	UniProt primary accession
PLOD2	Procollagen-lysine,2-oxoglutarate 5-dioxygenase 2	O00469
CES2	Cocaine esterase	O00748
TAX1BP3	Tax1-binding protein 3	O14907
INPP4B	Inositol polyphosphate 4-phosphatase type II	O15327
CLDN3;CLDN4;CLDN6;CLDN9	Claudin-3;claudin-4;claudin-6;claudin-9	O15551;O14493;P56747;O95484
KCNN4	Intermediate conductance calcium-activated potassium channel protein 4	O15554
SPINT2	Kunitz-type protease inhibitor 2	O43291
CYTH3	Cytohesin-3	O43739
SLIT3	Slit homolog 3 protein	O75094
TM7SF2	Delta-14-sterol reductase	O76062
UBL3	Ubiquitin-like protein 3	O95164
ADA	Adenosine deaminase	P00813
FGF1	Fibroblast growth factor 1	P05230
RBP1	Cellular retinol-binding protein 1	P09455
TPM1	Tropomyosin alpha-1 chain	P09493
PTPRF	Receptor-type tyrosine-protein phosphatase F	P10586
GYS1	Glycogen [starch] synthase, muscle	P13807
AKR1A1	Aldo-keto reductase family 1 member A1	P14550
ITGB5	Integrin beta-5	P18084
PTPRB	Receptor-type tyrosine-protein phosphatase beta	P23467
LOX	Protein-lysine 6-oxidase	P28300
EPHB2	Ephrin type-B receptor 2	P29323
FBN1	Fibrillin-1	P35555
GSK3B	Glycogen synthase kinase-3 beta	P49841
CAV2	Caveolin-2	P51636
EPHB3;EPHB4	Ephrin type-B receptor 3;ephrin type-B receptor 4	P54753;P54760
ACTA2	Actin, aortic smooth muscle	P62736
AKAP12	A-kinase anchor protein 12	Q02952
PLP2	Proteolipid protein 2	Q04941
PLCB4	1-phosphatidylinositol 4,5-bisphosphate phosphodiesterase beta-4	Q15147
TGFBI	Transforming growth factor-beta-induced protein ig-h3	Q15582
PTPN14	Tyrosine-protein phosphatase non-receptor type 14	Q15678
ECM1	Extracellular matrix protein 1	Q16610
ACTBL2	Beta-actin-like protein 2	Q562R1
MEST	Mesoderm-specific transcript homolog protein	Q5EB52
SYDE1	Rho GTPase-activating protein SYDE1	Q6ZW31
MBOAT2	Lysophospholipid acyltransferase 2	Q6ZWT7
CCDC80	Coiled-coil domain-containing protein 80	Q76M96
ATG9A	Autophagy-related protein 9A	Q7Z3C6
ORMDL3	ORM1-like protein 3	Q8N138
USP33	Ubiquitin carboxyl-terminal hydrolase 33	Q8TEY7
PPP1R13L	RelA-associated inhibitor	Q8WUF5
PALLD	Palladin	Q8WX93
RPE	Ribulose-phosphate 3-epimerase	Q96AT9

SLC35E1	Solute carrier family 35 member E1	Q96K37
ALS2	Alsin	Q96Q42
VKORC1	Vitamin K epoxide reductase complex subunit 1	Q9BQB6
DPCD	Protein DPCD	Q9BVM2
NDEL1	Nuclear distribution protein nudE-like 1	Q9GZM8
LTBP3	Latent-transforming growth factor beta-binding protein 3	Q9NS15
TCP11L1	T-complex protein 11-like protein 1	Q9NUJ3
ELOVL5	Very long chain fatty acid elongase 5	Q9NYP7
LMCD1	LIM and cysteine-rich domains protein 1	Q9NZU5
DIP2B	Disco-interacting protein 2 homolog B	Q9P265
SUN2	SUN domain-containing protein 2	Q9UH99
ERG28	Ergosterol biosynthetic protein 28 homolog	Q9UKR5
SLC4A7	Sodium bicarbonate cotransporter 3	Q9Y6M7
SPARC	Secreted protein acidic and cysteine rich	P09486
UBE2C	Ubiquitin-conjugating enzyme E2 C	O00762
COX7A2L	Cytochrome c oxidase subunit 7A-related protein, mitochondrial	O14548
RNF13	E3 ubiquitin-protein ligase RNF13	O43567
PRC1	Protein regulator of cytokinesis 1	O43663
TSPAN3	Tetraspanin-3	O60637
PTCD1	Pentatricopeptide repeat-containing protein 1, mitochondrial	O75127
CLDN11	Claudin-11	O75508
CREG1	Protein CREG1	O75629
SLC25A12	Electrogenic aspartate/glutamate antiporter SLC25A12, mitochondrial	O75746
KIF20A	Kinesin-like protein KIF20A	O95235
F13A1	Coagulation factor XIII A chain	P00488
GOT2	Aspartate aminotransferase, mitochondrial	P00505
CSTA	Cystatin-A	P01040
JCHAIN	Immunoglobulin J chain	P01591
PIGR	Polymeric immunoglobulin receptor	P01833
CRYAA	Alpha-crystallin A chain	P02489
KRT14	Keratin, type I cytoskeletal 14	P02533
LTF	Lactotransferrin	P02788
FTH1	Ferritin heavy chain	P02794
SOD2	Superoxide dismutase [Mn], mitochondrial	P04179
TK1	Thymidine kinase, cytosolic	P04183
KRT1	Keratin, type II cytoskeletal 1	P04264
ARG1	Arginase-1	P05089
APOD	Apolipoprotein D	P05090
S100A8	Protein S100-A8	P05109
RPLP2	Large ribosomal subunit protein P2	P05387
CRYBA1	Beta-crystallin A3	P05813
S100A9	Protein S100-A9	P06702
S100A6	Protein S100-A6	P06703
COL1A2	Collagen alpha-2(I) chain	P08123
FDX1	Adrenodoxin, mitochondrial	P10109
DLAT	Dihydrolipoyllysine-residue acetyltransferase component of pyruvate dehydrogenase complex, mitochondrial	P10515
TOP2A	DNA topoisomerase 2-alpha	P11388

PIP	Prolactin-inducible protein	P12273
CKMT1A	Creatine kinase U-type, mitochondrial	P12532
ACE	Angiotensin-converting enzyme	P12821
KRT10	Keratin, type I cytoskeletal 10	P13645
KRT5	Keratin, type II cytoskeletal 5	P13647
LCP1	Plastin-2	P13796
H1-5	Histone H1.5	P16401
ATP5PF	ATP synthase-coupling factor 6, mitochondrial	P18859
KRT15	Keratin, type I cytoskeletal 15	P19012
AZGP1	Zinc-alpha-2-glycoprotein	P25311
S100P	Protein S100-P	P25815
CRYBB3	Beta-crystallin B3	P26998
SERPINB3	Serpin B3	P29508
PRDX3	Thioredoxin-dependent peroxide reductase, mitochondrial	P30048
LCN1	Lipocalin-1	P31025
S100A7	Protein S100-A7	P31151
RRM2	Ribonucleoside-diphosphate reductase subunit M2	P31350
CASP14	Caspase-14	P31944
SDC2	Syndecan-2	P34741
SHMT2	Serine hydroxymethyltransferase, mitochondrial	P34897
PRSS3	Trypsin-3	P35030
KRT2	Keratin, type II cytoskeletal 2 epidermal	P35908
MDH2	Malate dehydrogenase, mitochondrial	P40926
HAL	Histidine ammonia-lyase	P42357
SLC1A4	Neutral amino acid transporter A	P43007
CRYBB2	Beta-crystallin B2	P43320
MRPL12	Large ribosomal subunit protein bL12m	P52815
CKS1B	Cyclin-dependent kinases regulatory subunit 1	P61024
LYZ	Lysozyme C	P61626
TIMM10	Mitochondrial import inner membrane translocase subunit Tim10	P62072
DCD	Dermcidin	P81605
MRPS15	Small ribosomal subunit protein uS15m	P82914
DSG1	Desmoglein-1	Q02413
SSBP1	Single-stranded DNA-binding protein, mitochondrial	Q04837
TGM3	Protein-glutamine gamma-glutamyltransferase E	Q08188
DSC1	Desmocollin-1	Q08554
SELENBP1	Methanethiol oxidase	Q13228
NME3	Nucleoside diphosphate kinase 3	Q13232
AAMP	Angio-associated migratory cell protein	Q13685
COX17	Cytochrome c oxidase copper chaperone	Q14061
DSC3	Desmocollin-3	Q14574
CDSN	Corneodesmosin	Q15517
CST6	Cystatin-M	Q15828
UBE2S	Ubiquitin-conjugating enzyme E2 S	Q16763
FLG2	Filaggrin-2	Q5D862
COA6	Cytochrome c oxidase assembly factor 6 homolog	Q5JTJ3
EEF1DP3	Putative elongation factor 1-delta-like protein	Q658K8
FDX2	Ferredoxin-2, mitochondrial	Q6P4F2
FGD6	FYVE, RhoGEF and PH domain-containing protein 6	Q6ZV73

KRT77	Keratin, type II cytoskeletal 1b	Q7Z794
MCAT	Malonyl-CoA-acyl carrier protein transacylase, mitochondrial	Q8IVS2
PHC2	Polyhomeotic-like protein 2	Q8IXK0
KRT78	Keratin, type II cytoskeletal 78	Q8N1N4
TMEM263	Transmembrane protein 263	Q8WUH6
MRPS27	Small ribosomal subunit protein mS27	Q92552
SORL1	Sortilin-related receptor	Q92673
ZG16B	Pancreatic adenocarcinoma up-regulated factor	Q96DA0
OMA1	Metalloendopeptidase OMA1, mitochondrial	Q96E52
SPDL1	Protein Spindly	Q96EA4
SERPINB12	Serpin B12	Q96P63
GSDMA	Gasdermin-A	Q96QA5
MED15	Mediator of RNA polymerase II transcription subunit 15	Q96RN5
PFDN5	Prefoldin subunit 5	Q99471
PKMYT1	Membrane-associated tyrosine- and threonine-specific cdc2-inhibitory kinase	Q99640
PYM1	Partner of Y14 and mago	Q9BRP8
PCDH11X;PCDH11Y	Protocadherin-11 X-linked;Protocadherin-11 Y-linked	Q9BZA7;Q9BZA8
KRT23	Keratin, type I cytoskeletal 23	Q9C075
RACGAP1	Rac GTPase-activating protein 1	Q9H0H5
RNASE7	Ribonuclease 7	Q9H1E1
MEGF9	Multiple epidermal growth factor-like domains protein 9	Q9H1U4
MRPL46	Large ribosomal subunit protein mL46	Q9H2W6
LYRM4	LYR motif-containing protein 4	Q9HD34
SARS2	Serine--tRNA ligase, mitochondrial	Q9NP81
UBE2T	Ubiquitin-conjugating enzyme E2 T	Q9NPD8
ANLN	Anillin	Q9NQW6
DIAPH3	Protein diaphanous homolog 3	Q9NSV4
MRPL22	Large ribosomal subunit protein uL22m	Q9NWU5
CALML5	Calmodulin-like protein 5	Q9NZT1
MTRES1	Mitochondrial transcription rescue factor 1	Q9P0P8
CYB5R1	NADH-cytochrome b5 reductase 1	Q9UHQ9
CPA4	Carboxypeptidase A4	Q9UI42
ACAD8	Isobutyryl-CoA dehydrogenase, mitochondrial	Q9UKU7
TIMM13	Mitochondrial import inner membrane translocase subunit Tim13	Q9Y5L4
